# Could Choice of Vehicles in Those with Attention Deficit Hyperactivity Disorder (ADHD) Contribute to This Group of Drivers’ Increased Accident Risk? A Questionnaire Study

**DOI:** 10.1192/bjo.2026.11136

**Published:** 2026-06-30

**Authors:** Simon Taylor

**Affiliations:** Derbyshire Healthcare NHS Foundation Trust, Chesterfield, United Kingdom

## Abstract

**Aims:**

ADHD drivers have up to four times higher risk of being involved in road traffic accidents than other drivers. Symptom severity has some influence on risk but comorbidity, especially for antisocial behaviours and alcohol misuse are strong associations with risk. It seems that ADHD drivers engage in more distracting tasks while driving and there is also an increased risk of sleep-related accidents. ADHD drivers tend to drive faster and it has been suggested that this is a strategy to escape the boredom.

This study tests the hypothesis that those with ADHD choose faster cars and that this may be another factor explaining this group’s higher accident risk. In addition, since those diagnosed with ADHD in childhood are more likely to take up motor sport, which is associated with increased risk of road collisions, this study also looked for an association of ADHD with engagement with motorsport in adult life.

**Methods:**

Participants, all employees at an NHS mental health trust, were invited to fill out an electronic questionnaire. A link was included within the weekly Trust newsletter. Questions covered demographic details, ADHD screening (ASRS), depression and anxiety screening (PHQ-4), two questions asking preference, if buying a car, between luxury and sports variants and three questions about motorsport interest. Research department advice, to use the NHS Health Research Authority’s “Is my study research?” decision tool, determined that the survey was not classified as research as results may not be generalisable. Power calculations suggested minimum n of 121.

**Results:**

In total 114 replies were received, 91 were female with a wide spread of age groups. Ninety-eight scored less than 6 on PHQ-4. Of these 27 scored on 4 or more items on ASRS, who were classified as ADHD group. More of the non-ADHD group chose the faster models, in fact for one of the two 31% chose the sportier version compared with only 7.4% of the ADHD group, p<0.05 (Chi squared). There was little difference in motorsport interest between groups. Of the 16 with higher PHQ-4 scores, 62% scored above ADHD cut-off.

**Conclusion:**

Contrary to the hypothesis, reasons why the ADHD group seemed to choose the more sedate cars are discussed in terms of the intriguing possibility that strategies adopted enabling management of responsible employment may influence other aspects of living. The importance of removing those with higher anxiety and depression scores from analysis is emphasised.

